# Response of beech and fir to different light intensities along the Carpathian and Dinaric Mountains

**DOI:** 10.3389/fpls.2024.1380275

**Published:** 2024-05-08

**Authors:** Matjaž Čater, Pia Caroline Adamič, Eva Dařenova

**Affiliations:** ^1^ Department of Yield and Silviculture, Slovenian Forestry Institute, Ljubljana, Slovenia; ^2^ Department of Silviculture, Faculty of Forestry and Wood technology, Mendel University, Brno, Czechia; ^3^ Department of Forestry and Renewable Forest Resources, Biotechnical Faculty, University of Ljubljana, Ljubljana, Slovenia; ^4^ Department of Department Of Matters And Energy Fluxes, Global Change Research Institute of the Czech Academy of Sciences, Brno, Czechia

**Keywords:** silver fir, beech, light response, Carpathian Mountains, Dinaric Mountains, temperature, precipitation

## Abstract

Predicting global change mitigations based on environmental variables, like temperature and water availability, although yielding insightful hypothesis still lacks the integration of environmental responses. Physiological limits should be assessed to obtain a complete representation of a species’ fundamental niche. Detailed ecophysiological studies on the response of trees along the latitudinal gradient are rare. They could shed light on the behaviour under different light intensities and other studied traits. The forests of the Dinaric Mountains and the Carpathians represent the largest contiguous forest complexes in south-eastern Europe. In uneven-aged Carpathian (8 plots) and Dinaric Mountain (11 plots) forests, net assimilation (A_max_) and maximum quantum yield (Φ) were measured for beech and fir in three predefined light intensity categories according to the indirect site factor (ISF%) obtained by the analysis of hemispherical photographs in managed and old growth forests, all located above 800 m a.s.l. The measurements were carried out under fixed environmental conditions in each light category per plot for three consecutive years. Data from the last 50-year average period from the CRU TS 4.01 dataset were used for the comparison between Amax, Φ, and climate. The highest Φ for beech were observed in the central part of the Dinaric Mountains and in the south westernmost and northwesternmost part of the Carpathians for both beech and fir, while they were highest for fir in the Dinaric Mountains in the northwesternmost part of the study area. The Φ-value of beech decreased in both complexes with increasing mean annual temperature and was highest in the open landscape. For fir in the Carpathians, Φ decreased with increasing mean annual temperature, while in the Dinaric Mountains it increased with higher temperature and showed a more scattered response compared to the Carpathians. Short-term ecophysiological responses of beech and fir were consistent to long-term radial growth observations observed on same locations. The results may provide a basis and an indication of the future response of two tree species in their biogeographical range to climate change in terms of competitiveness, existence and consequently forest management decisions.

## Introduction

Mixed fir-beech forests are an essential component of Central and South-Eastern European forest ecosystems and landscapes (Dobrowolska et al., 2017). Beech (*Fagus sylvatica* L.), the most common forest species in Europe (Ellenberg, 1988), grows in pure deciduous forests or in mixed forests with conifers, especially with silver fir (*Abies alba* Mill.) (hereafter fir), whose geographical distribution is comparable to that of beech, but is largely restricted to the Alpine and Carpathian arc (Bošela et al., 2018). Fir is the tallest tree in Europe and forms mixed forest stands in many regions (Aussenac, 2002; Dobrowolska et al., 2017). At its southern limit of distribution in the mountainous regions of the Iberian Peninsula and Italy, it forms mixed stands with Mediterranean tree species (Carrer et al., 2010). Species distribution modelling suggests that the current range of silver fir was determined by historical land use and which can mask the potential effects of climate change (Svenning and Skov, 2004; Tinner et al., 2013; Di Pasquale et al., 2014). However, there are several disagreements as to whether the recent increase in temperature alone or in combination with a reduction in precipitation will lead to a reduction in the species’ range (Alba-Sánchez et al., 2010; Maiorano et al., 2013). Dendroecological studies have shown a decline in the growth of silver fir in the Iberian Peninsula (Linares and Camarero, 2012) and in the south-eastern European mountains (Diaci et al., 2011), which is probably due to the increase in summer water deficit in these areas (Giorgi and Lionello, 2008). The resilience of plant species or populations depends on their ability to acclimatise to the new environmental conditions. Beech, on the other hand, shows an increase in abundance and a successful ability to regenerate after large-scale disturbances such as windthrow or sleat (Čater and Diaci, 2017; Čater, 2021).

Recent research has shown that the vegetation in the Carpathian forests is changing in different intensities and directions, which can be attributed to various processes (Šamonil and Vrška, 2007), such as air pollution, soil acidification (Hédl et al., 2011), and the competitive influence of tree seedlings (Łysik, 2009). Of particular interest is the question of how ecologically and economically valuable species such as silver fir will cope with recent climate trends (Maiorano et al., 2013; Tinner et al., 2013). Adamič et al. (2023) already confirmed different stem radial growth in beech and fir since 1950s and their response to climate conditions along the Carpathians, while Darenova et al. (2024) related soil respiration spatial variability with soil water content, soil carbon and nitrogen content and no significant affect connected with canopy gaps.

Forest gaps are an integral part of forest ecosystems and play a crucial role in the regeneration of mixed beech-conifer forests and influence the future species admixture (Grassi and Bagnaresi, 2001; Čater et al., 2014). Harvests alter micrometeorological stand’s conditions and ecological processes in the understorey. They result in higher soil temperature and precipitation throughfall, which temporarily increase soil respiration (Londo et al., 1999; Čater, 2021) and consequently increase the decomposition of soil organic matter. With reduced aboveground litter input, this leads to a loss of soil organic carbon (Hukić et al., 2021).

When predicting the effects of climate change on the future performance of tree species, a geographical, particularly a latitudinal gradient, can serve as a useful space-time proxy and might provide valuable reference to predict future limitations of these tree species (De Frenne et al., 2013; Čater and Levanič, 2019; Weithmann et al., 2022; Smith et al., 2023), also comparing responses in managed and old-growth forests. Many projections and predictions of global change impacts are based on theoretical specifications of temperature requirements and moisture/water availability for the life cycle of specific species (Dillaway and Kruger, 2010). Unfortunately, they do not take into account the actual response to conditions in the natural environment, which could provide a sufficient mechanistic basis for the exact nature of the constraints Physiological limits should be assessed to obtain a complete representation of a species’ fundamental niche and then constrain it with biotic interactions and effects of dispersal limitation (Meier et al., 2011).

In a study on the Balkan Peninsula along the Dinaric High Karst, where diverse and well-expressed ecological factors intertwine in a relatively short geographical distance (ca. 1000 km) (Bohn et al., 2000), the response of beech and fir from the southern, warmer and drier sites already successfully served as a highly probable future prediction for the same species response on currently less extreme sites in the north (Čater and Levanič, 2019). On the contrary are the Carpathian Mountains more complex and exhibit a sufficient latitudinal and longitudinal gradient associated with significant differences in temperature/precipitation as well as differences in seasonal patterns (Micu et al., 2016). Quantum yield (Φ) in various light microsites proved that beech is more efficient in exploiting direct radiation in sun exposed parts of the gap, compared to silver fir (Čater et al., 2014). Microsite position significantly influenced Φ (Lambers et al., 1998) of young beech and firs, which changed with gap size, explaining their difference in competitive ability (Čater et al., 2014). The abundance of microsite light categories along the elevation gradient in two silvicultural systems well indicated the forest structure and its fragmentation, and after large scale disturbances (Čater and Diaci, 2017; Čater, 2021), with quantum yield (Φ) as the resulting trait. Such division may also be associated with spatial distribution of other ecological factors: direct radiation may be related with an increased evapotranspiration and higher drought probability, while diffuse radiation with rainfall patterns within gaps (Krecmer, 1967).

Our aim was to compare the physiological responses of beech and fir along the geographical gradient of the Carpathian and Dinaric Mountains (1), to assess differences in the same light categories of both species between managed and old forests (2) and to verify the relationship between climatic parameters and ecophysiological traits of both species along both geographical gradients (3).

## Material and methods

### Research area

The Carpathians extend over a 1500 km long arc, the width of which varies from 170 km in the eastern and western parts to less than 80 km in the southern part of the mountain range (Warszyńska, 1995). The wide variety of favourable ecological conditions is reflected in the great diversity of plants and animals and the rich biodiversity (Mirek and Piekos-Mirkowa, 1992).

Situated on the edge of the Atlantic and continental climate regions, the western climate type with its anticyclonic weather pattern dominates over most of the Carpathians. A continental climate prevails on the eastern slopes of the Eastern Carpathians. In the foothills of the Western Carpathians the average air temperature in July is 19 ^0^C and in the Southern Carpathians 22 ^0^C. In the south-western part, the air temperature drops by 0.81 ^0^C per 100 metres difference in altitude and by 0.5 ^0^C in the south-eastern part of the Carpathians. Annual precipitation ranges from around 500 mm at the foothills of the Southern Carpathians to over 2000 mm on the peaks of the Tatra Mountains (Vološčuk, 1999) in the north. Flysch predominates in the eastern and outer Western Carpathians, crystalline and volcanic rocks in the inner band, while metamorphic rocks predominate in the Southern Carpathians (Rădulescu and Săndulescu, 1973; Golonka et al., 2018).

The Dinaric Mountain range stretches from the southern edge of the Eastern Alps in Slovenia to the mountain massif in North Macedonia; it is bordered by the Adriatic Sea to the west and the Pannonian Basin to the east (Gams, 1969). Most of the mountain range consists of Mesozoic rocks, mainly limestone and dolomite. The depth of the limestone and dolomite rocks is unique and is typically 1 to 3 kilometres, with considerable local variations (Gams, 1969). Westerly winds bring large amounts of moisture to the higher elevations along the western side of the mountain range. Precipitation at higher altitudes is relatively evenly distributed during the year, with snow cover often remaining for up to six months (Mihevc, 2010). The forest structure and composition in the region is strongly influenced by the interaction between the mountain relief, the karst area, the soils and the climatic gradient. The mountain forests above 800 metres include mainly beech-dominated forests and uneven-aged mixed forests, in which beech, fir and occasionally spruce occur to varying degrees. Large, forested areas inland have remained intact to this day and have been managed with low-intensity silvicultural systems for more than a century (Boncina et al., 2013), with several protected old-growth forest remnants scattered across the area.

**Figure 1:**
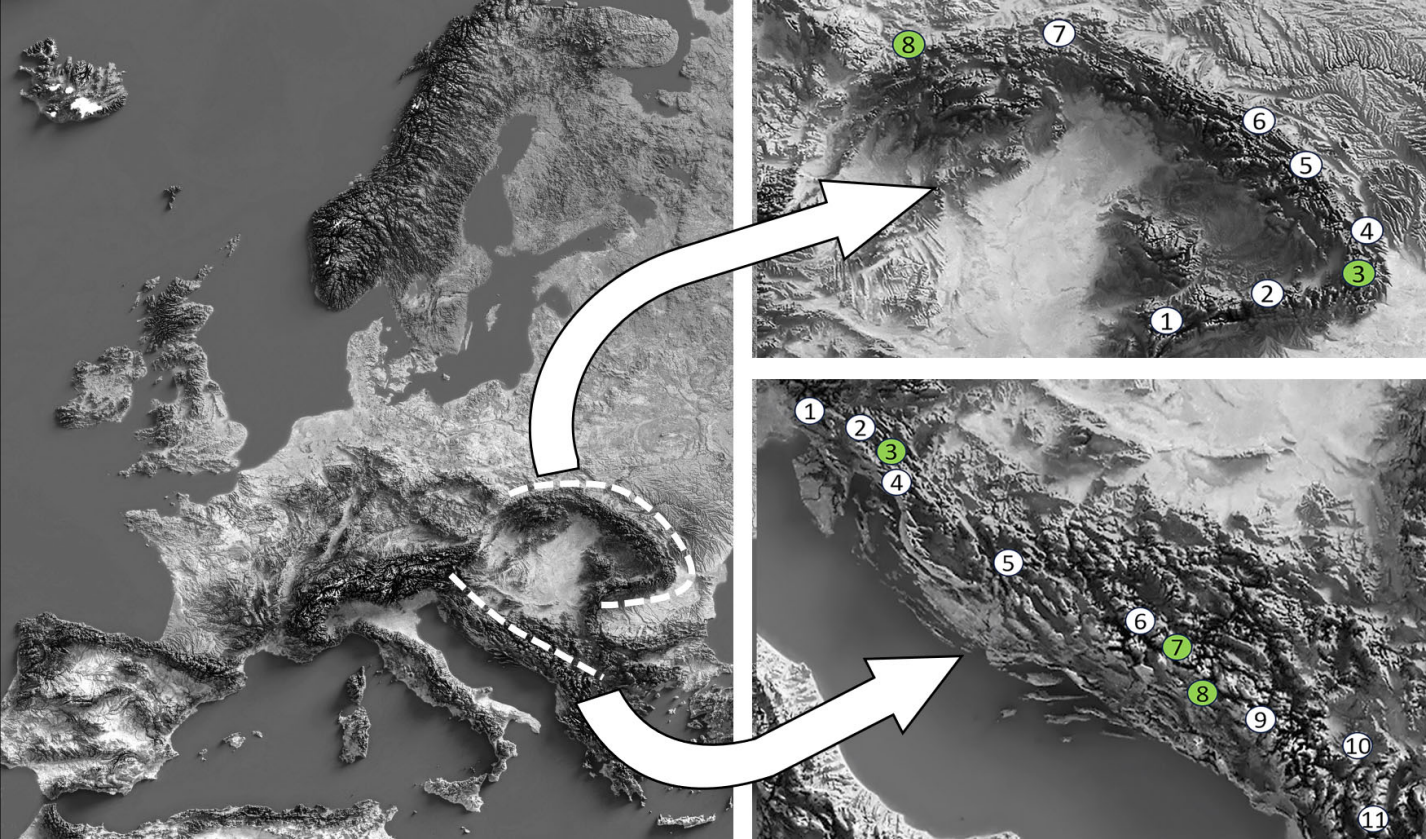
Research area and location of the permanent research plots along the Carpathian and Dinaric Mountains. The green dots represent old-growth reserves.

Selected permanent research plots were located above an altitude of 800 m a.s.l., where mature beech and fir trees predominate and where there is abundant natural regeneration of both tree species. Eight plots were established in the Carpathian Mountains, which extend from the Czech Republic from the far north-west through Slovakia and Romania to the southern part of the mountain range, and eleven permanent observation plots in the Dinaric Mountains, which extend from Slovenia from the far north-west through Croatia, Bosnia and Herzegovina and Montenegro along the mountainous region of the Balkan Peninsula to North Macedonia in the southern part of the mountain range. Two old stands were selected in the Carpathian Mountains (plots 3 and 8) and three in the Dinaric Mountains (plots 3, 7 and 8) (**Figure 1**; **Table 1**).

**Table 1:** Characteristics of the research plot; the meteorological data were obtained from the website “Climate Explorer” (http://climexp.knmi.nl) for the period 1985-2020, including the annual totals and the values for the April-September growing season.

Plot No/ Region		Altitude ASL (m)	Long. Deg (^0^)	Latit. Deg (^0^)	Annual precipitation (mm)	Average annual air T (^0^C)	Apr.-Sept. precipit. (mm)	Average April-Sept. air T (^0^C)
Carpathian Mts.	**1**	985	22.917°	45.169°	1073	4.7	695	10.7
	**2**	995	24.651°	45.460°	812	7.4	534	13.9
	***3***	1038	26.229°	45.614°	744	6.8	491	13.6
	**4**	830	26.604°	46.001°	603	8.3	412	15.6
	**5**	950	26.168°	46.854°	704	5.8	474	12.9
	**6**	850	25.683°	47.468°	738	5.4	501	12.0
	**7**	880	21.017°	49.255°	758	7.2	493	13.8
	***8***	820	18.417°	49.403°	744	7.1	491	13.4
Dinaric Mts.	**1**	814	13.757°	45.991°	1619	11.3	863	15.2
	**2**	807	14.464°	45.676°	1573	8.4	802	14.1
	***3***	871	15.004°	45.662°	1465	9.0	780	14.9
	**4**	1190	14.806°	45.271°	1108	9.3	616	14.9
	**5**	928	16.318°	44.307°	1349	8.6	645	14.5
	**6**	1204	18.269°	43.737°	1192	7.6	593	13.4
	***7***	1313	18.710°	43.320°	1229	7.7	607	13.4
	***8***	1408	18.646°	42.986°	1278	8.2	590	13.7
	**9**	1402	19.922°	42.553°	1163	6.6	548	13.1
	**10**	1410	20.885°	42.252°	850	8.6	418	14.6
	**11**	1315	20.741°	41.696°	836	8.4	357	14.0

### Potential light categories

In each plot, three categories of different light intensities were defined based on the analysis of hemispherical photographs, taken with the Canon EOS Rebel T3 DSLR and a calibrated fisheye lens with the Regent WinScanopy pro-d accessory. Young beech and fir are strongly influenced by indirect light, which has been confirmed by our former research (Čater et al. 2014); therefore, the Indirect Site Factor (ISF %) was used, describing relative share between potential indirect (diffuse) light at the point of measurement and in the open. At least eight hemispherical photographs per every plot were made before any response measurements (Čater and Levanič, 2013) in each of three potential categories: under a closed canopy with Indirect Site Factor (ISF)<15%, at the forest edge (15%<ISF<25%) and in the open ISF>25%. Colour digital hemispherical photographs were taken during windless weather and standard overcast sky conditions 150 cm above the forest floor when the solar disk was completely obscured. Exposure fitting was done to above canopy conditions prior to shooting on every plot (Macfarlane et al., 2000; Zhang et al., 2005) without noteworthy blooming effects (Leblanc et al., 2005). In the process of hemispherical photograph analysis, a ‘‘standard overcast sky’’ (SOC) model was applied for diffuse light distribution. For the calculation within the vegetation period (30. April - 31. September), the sun’s position was specified every 10 min. The solar constant was defined as 1,370 W/m^2^; 0.6 was set for atmospheric transmissivity and 0.15 for the proportion of diffuse radiation compared to calculated direct potential radiation. An automatic thresholding method based on the same colour scheme was applied for the discrimination between sky and canopy elements in all digital photographs, as the thresholding is crucial and may significantly affect the calculated parameters (Ishida, 2004; Nobis and Hunziker, 2005; Schwalbe et al., 2009).

### Weather and climate

**Figure 2:**
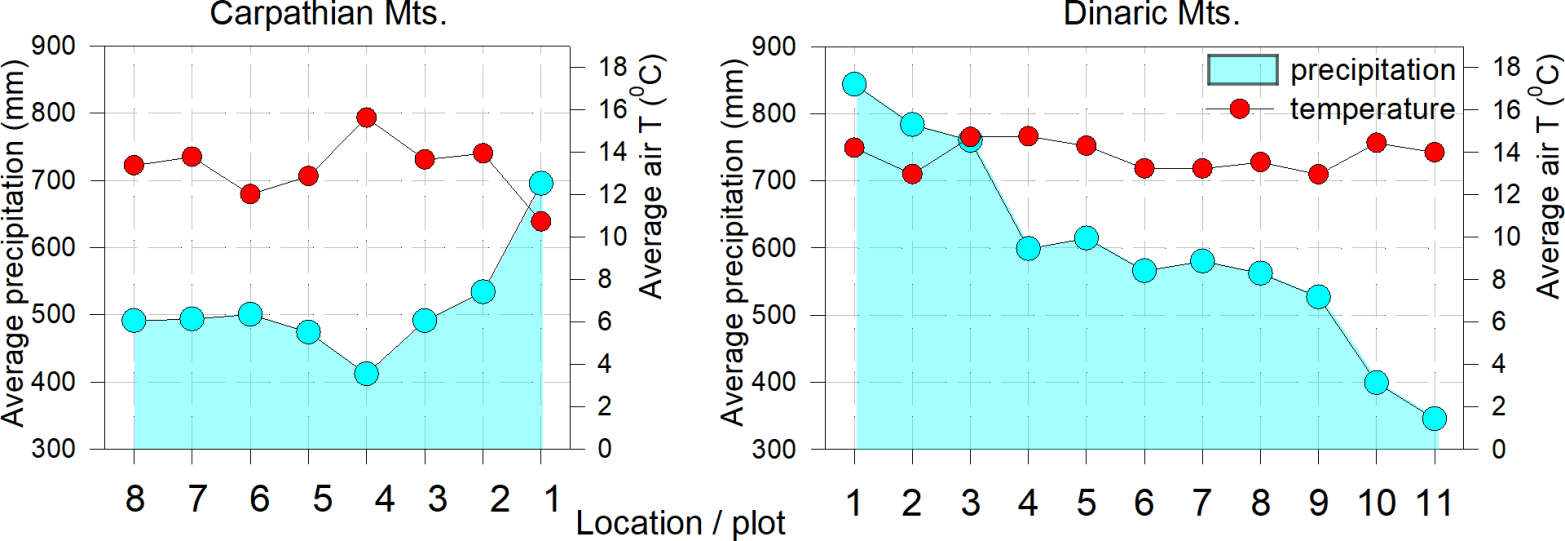
Average seasonal air temperature and precipitation (April-Sept., period 1951-2020) on the studied plots.

Monthly mean temperatures (°C) and monthly total precipitation data were interpolated for the 0.5° grids that include each selected plot and correspond to the CRU TS 4.01 dataset (Harris et al., 2014), obtained from the Royal Netherlands Meteorological Institute ‘Climate Explorer’ website (http://cliexp.knmi.nl). For the comparison between maximal net assimilation (A_max_), quantum yield (Φ) and climate data (temperature and precipitation), data from the last 50-year average period (1981-2020) were used. For the long-term comparison between climate and tree response, we extracted gridded climate data for mean monthly temperature and sum of monthly precipitation using the CRU TS 4.01 dataset with a resolution of 0.5x 0.5-degrees from the KNMI website (**Figure 2**).

### Nitrogen content and leaf mass per area

Leaves and needles were taken from the upper canopy position of at least 12 trees per light category and location and then stored in a cool, airtight place. Age of trees was similar in all three light categories. The same trees were also used to measure the maximum assimilation rate. Total leaf nitrogen concentration (N_tot_) [mg/g] was determined (Laboratory of Soil Science, Faculty of Forestry and Wood technology, Mendel University, Brno) to compare macronutrient status (Leco CNS-2000 analyser) (Lambers et al., 1998; Larcher, 2003) for the open, forest edge and closed canopy categories under mature trees (Čater et al., 2014). Fresh leaves were weighed and scanned for leaf area. Leaves were dried to constant weight at 105°C for 24 hours and weighed in the laboratory to determine leaf mass per area open-, forest edge- and closed canopy-category below mature trees (Čater et al., 2014). Leaves were dried to constant weight at 105°C for 24 hours and weighed in the laboratory to determine leaf mass per area (LMA) [g/m^2^].

### Assimilation light response

For the light saturation measurements, which were carried out in June and July in three consecutive growing seasons, at least 8 young trees of the same height that were not obstructed by their neighbours were randomly selected (*sensu* Čater and Levanič (2019). The age of the trees varied between 5-12 years. The light response was measured with a portable LI-6400 (Li-Cor, USA) system on at least four leaves/shoots per tree located in the upper third of the tree crowns. All assimilation values were recorded after they had held constant for 2 min or until the coefficient of variability (CV%) dropped below 5%.

• Light saturation curves were generated to compare net assimilation (A_max_) in young beech and fir trees under the same light conditions. All assimilation measurements were performed in the field at a constant temperature of the measurement block (20^0^C), a CO_2_ concentration of 420 µmol/l, an air flow of 500 µmols-1 and different light intensities: 0, 50, 250, 600 and 1500 µmol/m^2^/s. The maximum assimilation rates (A_max_) for the light saturation curves were used to compare the responses between different light categories and plots.
• The characteristic points of maximum quantum yield (Φ), defined as the maximum amount of fixed CO_2_ per amount of absorbed light quanta (Lambers et al., 1998) measured as the initial slope of the light response curve of CO_2_ fixation, were determined for each light category, species and plot, as described in Čater et al. (2014), using LiCOR software.

### Statistical analysis

Differences between the same years for the LMA, N_tot_, A_max_ and Φ were tested using two-way ANOVA with tree species (beech and fir) and light (open, edge, canopy) as dependent variables. Analyses of variance (ANOVA) and the HSD Tuckey *post-hoc* test were performed after testing data to meet conditions of normality. Probability values of p<0.05 (*), p<0.01 (**) and p<0.001 (***) were considered significant. Data analysis, correlation between the measured variables and multiple regression were performed using Statistica Data Analysis Software System (2011).

## Results

### Weather and climate

In both complexes, the long-term average temperatures show more homogeneous conditions over the longer Dinaric area and more variable conditions in Carpathians. The average annual precipitation in the Carpathian region is lower and corresponds to the conditions in the southern Dinaric mountains. (plot 9) (**Figure 2**).

### Foliar nitrogen

In all plots, N_tot_ was highest for both beech and fir in the open and lowest in the closed canopy, without significant differences between light categories and years. On all studied plots N_tot_ was within the optimal thresholds 13-15mg/g for fir and 18-22mg/g for beech, as reported by Grassi and Bagnaresi (2001); Mellert and Göttlein (2012) or even above range reported by Yang et al. (2022) and Bachofen et al. (2020). The same trend was observed for LMA.

**Figure 3:**
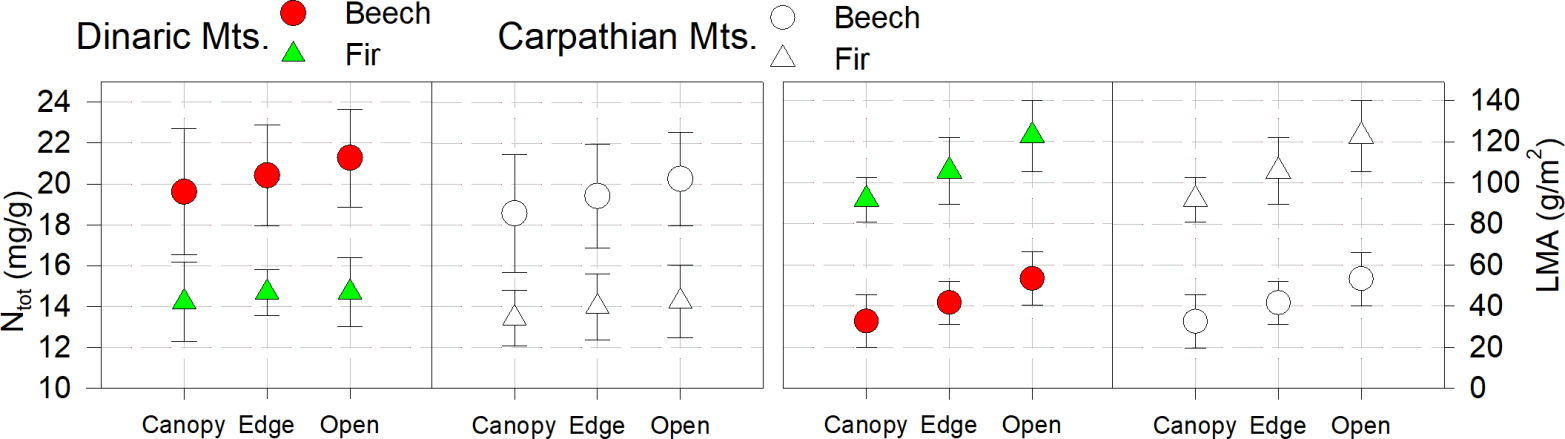
Foliar nitrogen (N_tot_) and leaf mass per area (LMA); bars are standard errors.

The values of N_tot_ and LMA were slightly and not significantly lower in all categories in the Carpathian Mountains than in the Dinaric Mountains (**Figure 3**, **Table 2**).

**Table 2:** ANOVA for leaf nitrogen (N_tot_) and leaf mass per area (LMA) in both regions.

Region	Trait	Df 1;2	Species		Df 1;2	Light category		Df 1;2	Species X Light category	
			F	p		F	p		F	p
Carpathian Mts.	N_tot_	1; 47	176.2	2e-17***	2; 43	4.2	0,0278*	2; 43	1.8	0.5731**^NS^**
	LMA	1; 47	3215.4	2e-17***	2; 43	345.4	2e-17***	2; 43	28.2	2e-17***
Dinaric Mts.	N_tot_	1; 65	193.2	2e-16***	2; 60	3.6	0,0336*	2; 60	0.7	0.5197**^NS^**
	LMA	1; 65	5859.9	2e-16***	2; 60	415.1	2e-16***	2; 60	46.0	7.6e-13***

p<0.05 (*), p<0.001 (***), ns – non significant.

### Maximum assimilation rate and quantum yield (Φ)

**Figure 4:**
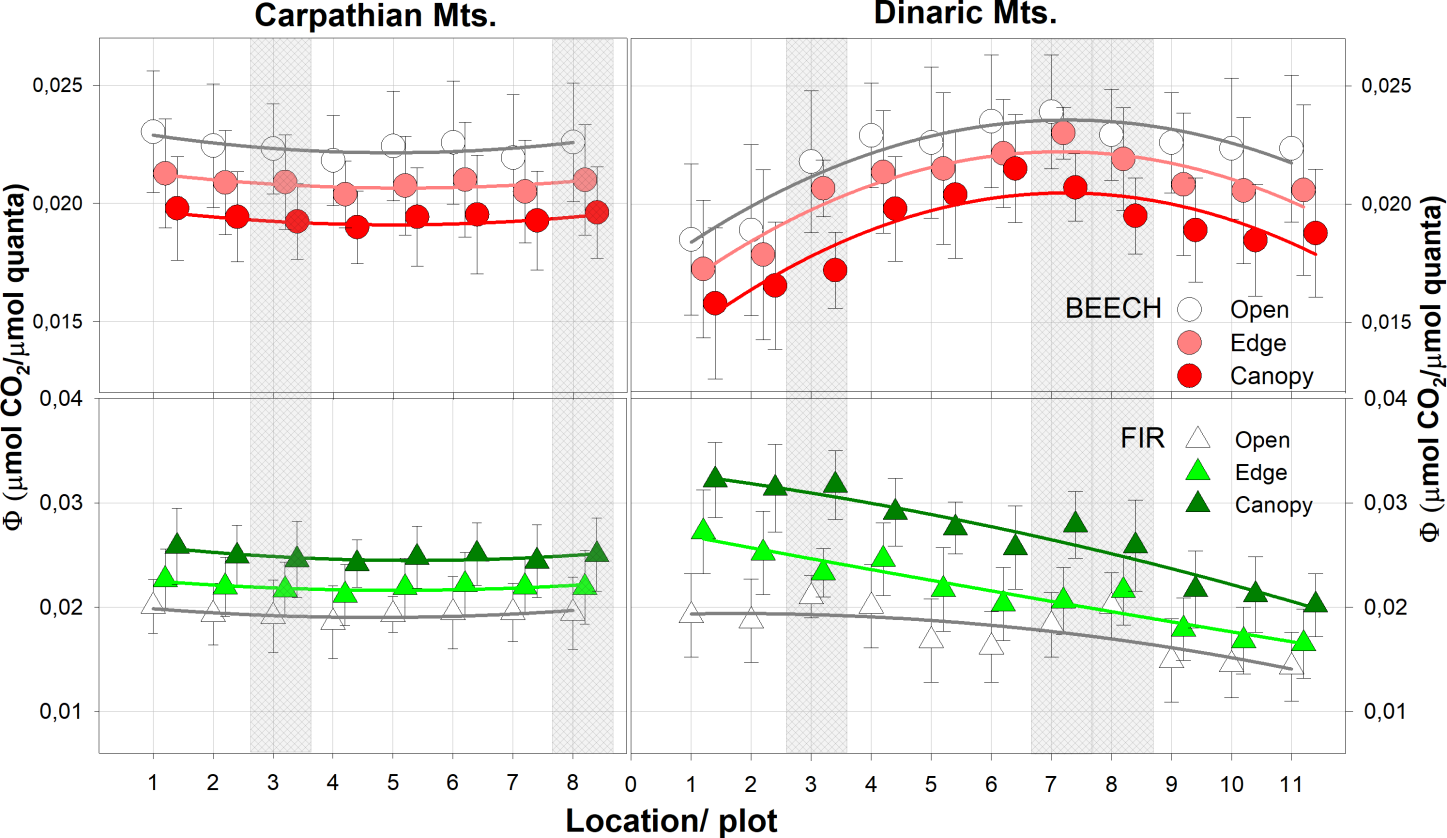
Average quantum yield (Φ) in all light categories. The shaded areas represent old growth reserves. N for each light category = 24.

In the Carpathian and Dinaric Mountains, Φ followed the pattern of precipitation and temperature; in both complexes it was highest for beech in the open light and for fir under closed canopies. In old growth reserves of the Dinaric Mountains, Φ was shifted towards the response of open light category for both species, much more so than in the Carpathian Mountains. In all cases, the absolute values were higher in all light categories than in the neighbouring managed forest stands (**Figure 4**, **Table 3**).

**Table 3:** ANOVA for maximum assimilation rate (A_max_) and quantum yield (Φ) for beech and fir in different light conditions and complexes.

Complex	Trait	Df 1;2	Species		Df 1;2	Light category		Df 1;2	Species X Light category	
			F	p		F	p		F	p
Carpathian Mts.	**A_max_**	1; 1096	1783.5	2e-17***	2; 1096	1476.4	2e-17***	2; 1096	987.5	2e-17***
	**Φ**	1; 1096	622.2	2e-17***	2; 1096	214.9	2e-17***	2; 1096	2869.5	2e-17***
Dinaric Mts.	**A_max_**	1; 1578	1454.3	2e-16***	2; 1578	1352.3	2e-16***	2; 1578	89.53	2e-15***
	**Φ**	1; 1578	73.0	2e-16***	2; 1578	231.0	2e-16***	2; 1578	775.4	2e-15***

p<0.001 (***).

The highest values (Φ) for beech were observed in the central part of the Dinaric Mountains region and in the south westernmost and northwesternmost part of the Carpathian Arc for both beech and fir, while Φ for fir was highest in the Dinaric Mountains in the northwesternmost part of the studied area (**Figure 4**).

*Post-hoc* analyses revealed significant differences between all light categories for Φ in Dinaric Mountains, except in the old-growth reserves, where no significant differences between forest edge and open light were confirmed for either species. In Carpathian Mountains differences between light categories were not so pronounced (**Table 4**).

**Table 4:** *Post hoc* (HSD) analysis for quantum yield (Φ) for beech and fir between C (canopy), E (edge) and O (open) light conditions.

Plot No/		Beech			Fir		
Region		C-E	C-O	E-O	C-E	C-O	E-O
Carpathian Mts.	**1**	*	***	*	*	***	*
	**2**	*	***	*	*	***	*
	***3***	*	***	ns	*	***	ns
	**4**	*	***	*	*	***	*
	**5**	*	***	*	*	***	*
	**6**	*	***	*	*	***	*
	**7**	*	***	*	*	***	*
	***8***	*	***	ns	*	***	ns
Dinaric Mts.	**1**	***	***	**	***	***	***
	**2**	***	***	*	***	***	***
	***3***	***	***	ns	***	***	ns
	**4**	***	***	**	***	***	***
	**5**	***	***	**	***	***	***
	**6**	ns	***	***	***	***	**
	***7***	***	***	ns	***	***	ns
	***8***	***	***	ns	***	***	ns
	**9**	***	***	***	***	***	*
	**10**	***	***	***	***	***	*
	**11**	***	***	***	***	***	*

Probability values: p<0.05 (*), p<0.01 (**), p<0.001 (***), ns – non significant.

**Figure 5:**
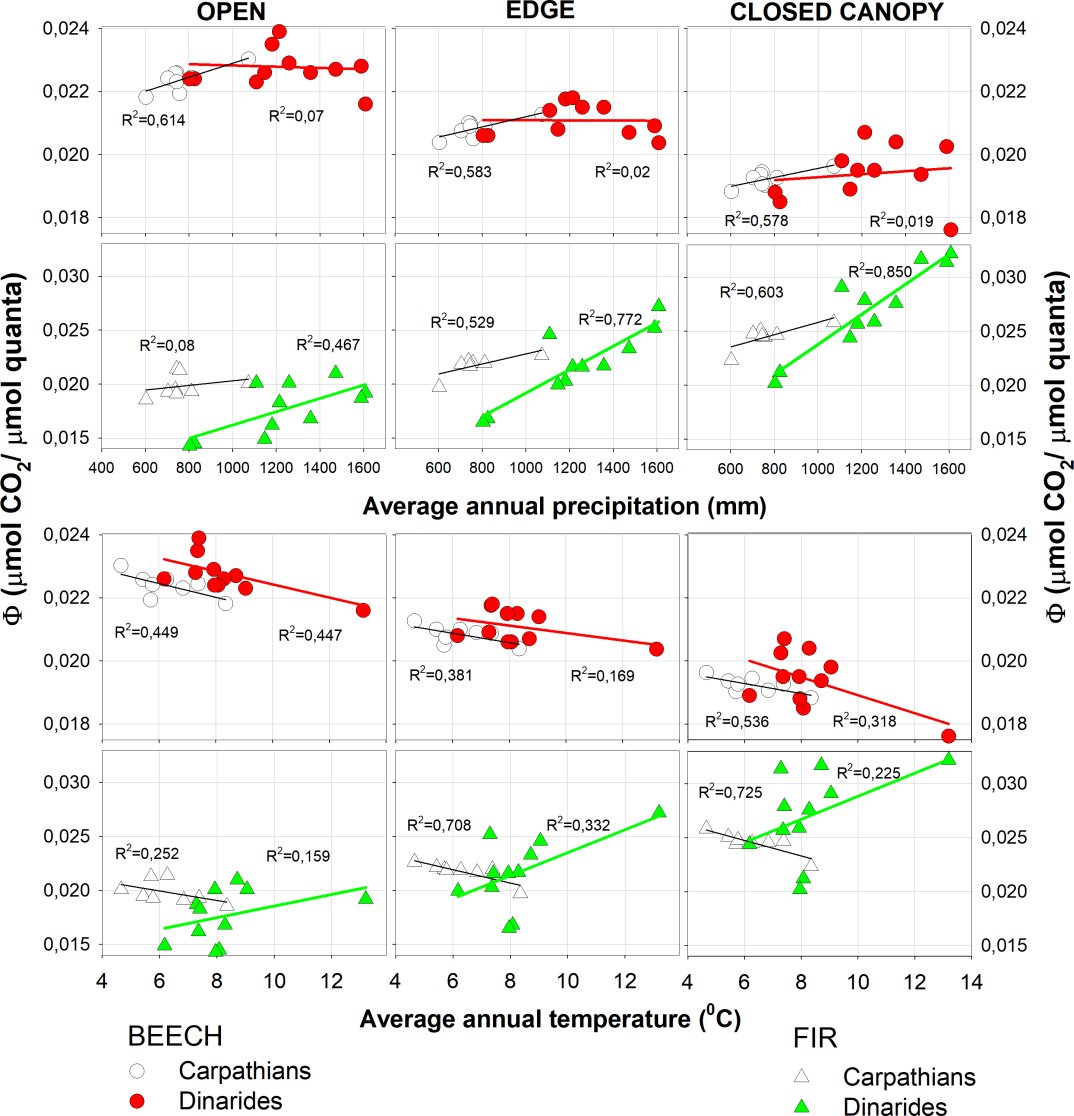
Quantum yield (Φ) as a function of precipitation and temperature.

We confirmed positive correlation between Φ and annual precipitation, which increased with the light intensity for beech in all light categories and in both the Carpathian and Dinaric Mountains. The correlation was positive for fir, decreased with increasing light and was highest when the canopy was closed. Slope of the curve for fir was steepest for the closed canopies (**Figure 5**).

The quantum yield of beech in both complexes decreased with increasing mean annual temperature and was highest in the open. For fir in the Carpathian Mountains, Φ decreased with increasing mean annual temperature, while in the Dinaric Mountains it increased with higher temperature and showed a more scattered response compared to the Carpathian Mountains (**Figure 5**).

**Figure 6:**
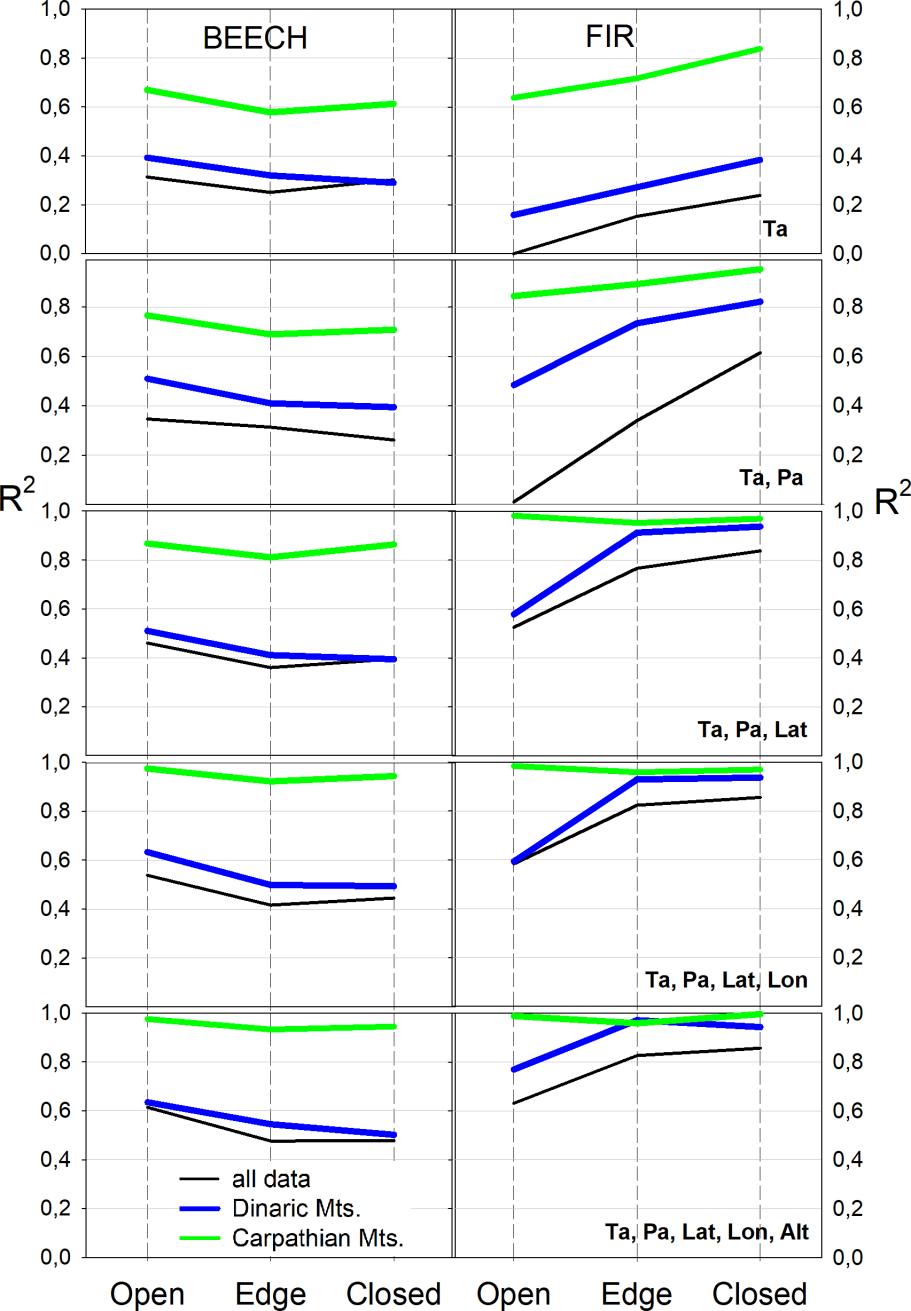
Multiple regression coefficients between independent (average annual air temperature - Ta, average annual precipitation - Pa, latitude - Lat, longitude - Lon, altitude - Alt) and dependent quantum yield (Φ) variables for both species, three light categories and two complexes.

The relationship between Φ and five independent parameters (latitude, longitude, average annual air temperature, annual precipitation, and altitude) was tested in a linear multiple regression model for both species, both complexes and three potential light categories. As the number of variables increased, the regression coefficients for beech in the Carpathians and Dinaric Mountains became increasingly different; beech in Dinaric Mountains indicated strongest relation with annual temperatures and additional parameters did not increase correlations as much as for the beech in Carpathian Mountains. The regression coefficients for fir between Dinaric and Carpathian Mountains were at first different and highest in Carpathian Mountains and became with additional parameters increasingly similar (**Figure 6**).

## Discussion

Shifts in climate zones and changes in forest cover directly affect regional surface temperatures through the exchange of water and energy; as warming continues, the frequency, intensity, and duration of heat-related events, including heat waves, are expected to increase (Lee et al., 2023). Climate zones are projected to shift further poleward in the mid- and high-latitudes and forest disturbances such as drought, wildfires and pest infestations are projected to increase (Lee et al., 2023). Some spatial distribution models predict a reduction in the ranges of fir and beech forests by 2100 due to climate change in favour of more drought-tolerant species (Piedallu et al., 2013); however, several studies question the predominant effects of ecological rather than macroecological and phytogeographical gradients on vegetation (Willner, 2002; Marinšek et al., 2013).

The selected sites in both studied mountain complexes were above 800m a.s.l. to ensure comparable and similar climatic conditions. Altitude is the key factor controlling the microclimate in temperate mountain forest stands (Körner et al., 2016). The average annual temperatures at the selected Carpathian sites ranged between 12 and 14 ^0^C with the exception of sites 4 and 1, while the average annual temperatures at the Dinaric sites showed more homogeneous conditions (13 to 14 ^0^C). The average amount of precipitation in the Dinaric Mountains decreased evenly from the north-west to south-east, while the amount of precipitation in the Carpathians decreased from west to east.

### Assimilation response

In the Dinaric Mountains, Φ was highest for beech in the central area (Bosnia and Herzegovina) and for fir in the north-western part of the transect (Slovenia, Croatia), while in the Carpathians it was highest at the beginning and end of the studied transect, at the westernmost sites.

The responses (Φ) of the two species studied between the Carpathians and the Dinaric Mountains in the same light categories showed certain similarities: in both cases, Φ values were higher for beech in the open, and lowest under shaded canopies, and vice versa for fir - highest under shaded conditions and lowest in the open, confirming our previous studies (Čater and Levanič, 2013, 2019).

The differences between old-growth forest reserves and neighbouring managed forests in both mountain complexes showed the same, higher values in all light categories and species, although the response was less pronounced in the Carpathians than in the Dinaric Mountains (**Figure 4**). It is not clear what caused the shift of Φ in the edge light category in the old growth reserves towards the open category for both beech and fir, as leaf nitrogen values were comparable between sites and were in the optimal range on all plots. In old growth reserves Amax and Φ were significantly higher than in managed forests due to microclimate, relative air humidity (RH), higher water use-efficiency (WUE) and photosynthetic nitrogen use efficiency (PNUE) in old growth reserves (Čater and Levanič, 2013).

Despite the non-significantly lower amount of foliar nitrogen (N_tot_) in the Carpathian categories, the variability of Φ and the differences between light categories were much higher for both beech and fir in the Dinaric Mountains (**Table 3**), possibly reflecting the more diverse growing conditions and more abundant water availability in the Dinaric Mountains than in the Carpathians (Micu et al., 2016). We assume that water is the most important limiting parameter, as the response of both species at all study sites in the Carpathians, except the first two, corresponded to the conditions in the lower south-eastern part of the Dinaric Mountains, where the average annual precipitation was below 500 mm (**Figures 2**, **4**).

With increasing precipitation, Φ increased in both fir and beech in the Carpathian and Dinaric Mountains, especially in fir, where the slope and dependence on shading increased. The correlation between Φ and mean annual temperature was negative at all sites for beech and fir in the Carpathians, while in the Dinaric Mountains the correlation was reversed for fir (**Figure 5**). The main reason for the negative correlation between Φ and increasing mean temperature in all light categories for fir could be the lower precipitation in the Carpathians.

Precipitation may not be the only reason, as the Eastern Carpathians and the southern Dinaric Mountains represent the edge of the silver fir’s natural range (Mauri et al., 2016; Caudullo et al., 2017). In south-eastern Europe, a higher resistance (compared to other tree species) to climate extremes was found (Bošela et al., 2018). At the same time, two populations of fir trees were distinguished in the Carpathians (Bošela et al., 2016): the eastern population reacts to drought similarly to the populations in the Balkan region, while the western population seems to be less affected by summer droughts. The fir of the western population might therefore be better adapted to the conditions of the Western Carpathians (Bošela et al., 2018) than the beech; although we observe a long-term expansion of beech and a decline of fir there for other reasons - see Vrška et al. (2009) and might also be better adapted to climate change than the eastern fir population.

The value of the correlation coefficient of the multiple regression increased with increasing number of independent variables and Φ for the beech in the Carpathians, while it remained the same for the Dinaric Mountains, which could indicate that the independent variables influence the beech differently in both mountain complexes (**Figure 6**, left). Fir, on the other hand, showed a similar dependence on an increasing number of the same independent variables in both the Carpathians and the Dinaric Mountains.

Beech can tolerate a wide range of light conditions in the understorey and manages to grow under different light conditions at young growth stages (Collet et al., 2001; Stancioiu and O’Hara, 2006; Nicolini and Caraglio, 2011). Fir, on the other hand, is a late successional species that is more shade-tolerant and more sensitive to water deficits than beech (Rolland et al., 1999). Compared to beech, the competitive ability of fir is much greater under low and diffuse light conditions, but consequently lower under medium and extensive light conditions; in gaps, beech adapts better and faster to rapid changes in light intensity (Lichtenthaler et al., 2007; Wyka et al., 2007; Čater et al., 2014), while the acclimation of fir growth rate to light conditions occurs gradually over several years (Robakowski et al., 2004). Our study confirmed a better light utilisation of fir in the shade and a different relationship with increased average temperatures in combination with lower precipitation. The lower Φ of fir utilisation of high-intensity solar radiation compared to beech could be a competitive disadvantage in large gaps in the canopy, which could limit the recruitment of species in the understorey or in small gaps, especially when mixed with beech.

### Paralleling short term assimilation with the growth response

The dependence of tree growth on precipitation has increased over the last century, and there has been an upward trend in drought since the 1950s. The latitudinal progression of radial growth decline and the proportion of positive trends indicate a rapid northward movement of the Mediterranean climate due to global changes and their impact on tree ecology (Gazol et al., 2015). The comparison of the assimilation response in young beech and fir trees was in good agreement with the growth response in adult trees (Čater and Levanič, 2019).

The study of Adamič et al. (2023) confirmed clear differences in the growth response to climate (temperature and precipitation) between southern, eastern and northern locations on the same study plots: a significant correlation between tree growth of both species and seasonal variables (temperature, precipitation) was observed on the eastern Carpathian sites (plots 4, 5 and 6), and a less or non-significant correlation in the southern sites (plots 1, 2 and 3). The fir in the north (plot 7) showed even less significant correlations than those at the southern sites, while the beech in the north showed more significant correlations than at the southern sites, but less than at the eastern sites. Beech and fir showed the same significant correlations at the eastern sites, while fir showed slightly more significant correlations in the south (Adamič et al., 2023). Accordingly, the quantum yield of beech and fir showed the lowest values in the eastern part and the highest values in the west.

Our research in the Dinaric Mountains confirmed that the growth of fir responded more strongly to climate than that of beech in the same study plots, as shown in this study (Čater and Levanič, 2019). Both temperature and precipitation had a stronger influence on the growth of fir than on that of beech. The climate signal of fir became weaker from NW to SE, with only the drought indices remaining significant, while the response of beech to climate was weaker in all plots and decreased from NW to SE, similar to fir (Čater and Levanič, 2019). In the Dinaric Mountains, four different groups were formed according to similar growth responses: two northern regions - A (including plots 1-4) and B (plot 5), and two southern regions - C (plots 6-8) and D (plots 9-11). The average Φ corresponded well to the growth response of the same group (A, B, C, D) and was more pronounced for the longer latitudinal distance than for the Carpathian Arc, which is similar in distance but shorter in latitudinal scale.

In the future, above-average summer temperatures and the absence of summer precipitation (July) are expected to become more frequent (Adamič et al., 2023), which could influence the future demography of fir towards the north and higher altitudes (Tinner et al., 2013). Especially in the Carpathian Mountains, which already show a negative correlation between young fir trees and increasing temperatures (**Figure 5**), the increasing number of extreme weather events is likely to affect young fir regeneration. The reaction of beech at the expense of fir and its spread in Central Europe has already been reported (Šamonil et al., 2009; Vrška et al., 2009; Janík et al., 2014, 2016).

Recent studies describe different responses of fir along its range (Diaci et al., 2011), its disappearance from warmer and drier areas and at the limit of its range (Ficko et al., 2011) and in south-western Europe (Gazol et al., 2015), especially in the Mediterranean region, where the decline of fir is often related to increasing drought (Čavlović et al., 2015). A higher resistance (compared to other tree species) to climate extremes was also found in south-east Europe (Bošela et al., 2018).

Studies also indicate a different response of the species along its distribution range. The radial growth of silver fir has increased significantly in Central Europe over the last 30 years, while it has decreased in drought-prone Mediterranean regions (Büntgen et al., 2014). Furthermore, different growth patterns have been observed between northern and southern populations of silver fir in Italy (Carrer et al., 2010).

Understanding the light utilisation processes in the regeneration phase of uneven-aged forests, which represent the largest contiguous forest complexes in south-eastern Europe, and focusing on sites that already have lower resilience to increasing temperatures and lower precipitation could help to maintain and emphasise the necessary measures that would contribute to greater stability and ensure continuous forest cover in the long term (Schütz, 2002; Schütz et al., 2016). The future response of silver fir forests to climate warming is currently being debated by the ecological community, as millennia of human impact have greatly reduced the species’ geographic distribution (Tinner et al., 2013; Di Pasquale et al., 2014). As the severity of disturbances is increasing and several Central European countries are facing unprecedented events (Nagel et al., 2017), the disadvantages of uneven-aged forest management include the dependence on shade-tolerant species that may be affected by the climatic conditions of the open areas created by disturbances. The most important silvicultural tool for the indirect promotion of silver fir is the creation of appropriately large gaps in the canopy and their temporal and spatial expansion. Most studies pointed to the predominance of fir under relatively closed canopies (Hohenadl, 1981; Stancioiu and O’Hara, 2006) and focused on different growth patterns without considering the ecophysiological processes involved.

In the present study, relatively short-term ecophysiological responses of beech and fir provided information on the behaviour at three different light intensity categories compared to long-term radial growth observations, which were consistent. The efficiency of beech increased with light intensity in all light categories and in both mountain complexes, while the response of fir was the opposite, decreasing with increasing light. The main difference between the two larger areas was the response of young fir to increasing temperatures, which correlated positively with increasing temperatures in the Dinarides and negatively in the Carpathians. In our opinion, this difference is related to the high precipitation in the Dinaric Mountains and the low precipitation in the Carpathians.

Our results may give an indication of how two important tree species in their biogeographical range will react to climate change in the future, which will affect their competitiveness, their existence and, consequently, forest management decisions.

## Data availability statement

The raw data supporting the conclusions of this article will be made available by the authors, without undue reservation.

## Ethics statement

No plants or animals (including human) were harmed during this study.

## Author contributions

MC: Conceptualization, Data curation, Formal Analysis, Funding acquisition, Investigation, Methodology, Project administration, Resources, Supervision, Validation, Visualization, Writing – original draft, Writing – review & editing. PA: Investigation, Writing – review & editing. ED: Investigation, Visualization, Writing – review & editing.
